# Incidence of stillbirth and perinatal mortality and their associated factors among women delivering at Harare Maternity Hospital, Zimbabwe: a cross-sectional retrospective analysis

**DOI:** 10.1186/1471-2393-5-9

**Published:** 2005-05-05

**Authors:** Shingairai A Feresu, Siobán D Harlow, Kathy Welch, Brenda W Gillespie

**Affiliations:** 1 Department of Preventive and Societal Medicine, University of Nebraska Medical Center 984350 Nebraska Medical Center, Omaha, NE 68198-4350, USA; 2 Department of Epidemiology, School of Public Health, University of Michigan, 109 Observatory Rd, Ann Arbor, MI 48105, USA; 3 Department of Community Medicine, University of Zimbabwe, Box A178, Avondale, Harare, Zimbabwe; 4 Center for Statistical Consultation and Research, University of Michigan, 3554 Rackham Building, 915 E Washington St, Ann Arbor, MI 48109-1070, USA

## Abstract

**Background:**

Death of an infant in utero or at birth has always been a devastating experience for the mother and of concern in clinical practice. Infant mortality remains a challenge in the care of pregnant women worldwide, but particularly for developing countries and the need to understand contributory factors is crucial for addressing appropriate perinatal health.

**Methods:**

Using information available in obstetric records for all deliveries (17,072 births) at Harare Maternity Hospital, Zimbabwe, we conducted a cross-sectional retrospective analysis of a one-year data, (1997–1998) to assess demographic and obstetric risk factors for stillbirth and early neonatal death. We estimated risk of stillbirth and early neonatal death for each potential risk factor.

**Results:**

The annual frequency of stillbirth was 56 per 1,000 total births. Women delivering stillbirths and early neonatal deaths were less likely to receive prenatal care (adjusted relative risk [RR] = 2.54; 95% confidence intervals [CI] 2.19–2.94 and RR = 2.52; 95% CI 1.63–3.91), which for combined stillbirths and early neonatal deaths increased with increasing gestational age (Hazard Ratio [HR] = 3.98, HR = 7.49 at 28 and 40 weeks of gestation, respectively). Rural residence was associated with risk of infant dying in utero, (RR = 1.33; 95% CI 1.12–1.59), and the risk of death increased with increasing gestational age (HR = 1.04, HR = 1.69, at 28 and 40 weeks of gestation, respectively). Older maternal age was associated with risk of death (HR = 1.50; 95% CI 1.21–1.84). Stillbirths were less likely to be delivered by Cesarean section (RR = 0.64; 95% CI 0.51–0.79), but more likely to be delivered as breech (RR = 4.65; 95% CI 3.88–5.57, as were early neonatal deaths (RR = 3.38; 95% CI 1.64–6.96).

**Conclusion:**

The frequency of stillbirth, especially macerated, is high, 27 per 1000 total births. Early prenatal care could help reduce perinatal death linking the woman to the health care system, increasing the probability that she would seek timely emergency care that would reduce the likelihood of death of her infant in utero. Improved quality of obstetric care during labor and delivery may help reduce the number of fresh stillbirths and early neonatal deaths.

## Background

Perinatal mortality remains a challenge in the care of pregnant women worldwide, particularly in developing countries [1-3]. To address the problem of perinatal mortality, factors associated with stillbirth, a major contributor of over 50% of perinatal deaths in developing countries, [4] must be understood. Stillbirths are both common and devastating, and in developed countries, about one third has been shown to be of unknown or unexplained origin [4,5]. As is the perinatal mortality rate, the stillbirth ratio is an important indicator of the quality of antenatal and obstetric care [2,3,6], but studies have not distinctively differentiated the frequency of and risk factors for macerated versus fresh stillbirths. Understanding the distribution of fresh and macerated stillbirths and deaths within the immediate postpartum period may help identify the quality of antenatal and obstetric care available to the pregnant women and prioritize appropriate intervention strategies. Macerated stillbirths are often associated with insults that occur in utero during the antenatal period, while fresh stillbirths and early neonatal deaths or mortality (ENNM) may suggest problems with the care available during labor and at delivery [3,7,8]. Few studies from Zimbabwe [9-11], have examined frequency of perinatal mortality and how this outcome varies across important demographic subgroups. Studies from developing countries have not considered the frequency of macerated and fresh stillbirths and their relationship to preterm birth or low birth weight (LBW) [1], and no such study has been conducted in Zimbabwe.

In Zimbabwe, perinatal mortality remains unacceptably high. In Harare, the capital city, perinatal mortality declined from 83 per 1,000 live births in 1978, to 34 per 1,000 live births in1984 and has changed little since then [12,13]. In 1983, an audit of all births occurring within the Greater Harare Maternity Unit (GHMU), which comprises of Harare Maternity Hospital (HMH) and the 12 municipal clinics in Harare, estimated perinatal mortality to be 34.5 per 1,000 live births, with preterm birth being the leading cause of perinatal mortality, accounting for 19.3% of perinatal deaths [14]. By 1989, perinatal mortality had risen to 47 per 1,000 live births [12,13]. Iliff and Kenyon [12,13] estimated that an increase in the number of and mortality from preterm births accounted for about half this increase. In the same study, stillbirth ratio was estimated to be 26 per 1,000 total births. A more recent study estimated the frequency of stillbirth at HMH to be 57 per 1,000 total births [9], which, using conservative assumptions, translates to 33 per 1,000 total births for the GHMU. Prevention of perinatal deaths is critical, especially those associated with LBW and preterm birth, since intuitively, infants who are born early or small have increased risk of morbidity and mortality [2,3,9,14].

Data on the frequency and distribution of adverse birth outcomes are important for planning maternal and child health care services in developing countries, and knowledge of local patterns of morbidity and mortality is essential for improving antenatal and obstetric care. Ensuring a safe and healthy delivery for both mother and child is a priority of the Zimbabwe health care delivery system and is an essential component of safe motherhood initiatives. In this preliminary study, we assessed the contribution of socio-demographic and reproductive/obstetric risk factors to the frequency of fresh and macerated stillbirth and ENNM over a one-year period at HMH, in Zimbabwe.

## Methods

The study was carried out at HMH, the largest referral hospital in Zimbabwe. The study was approved by the University of Michigan Institutional Review Board and the Medical Research Council of Zimbabwe, and permission to conduct the study was obtained from the Ministry of Health in Zimbabwe, HMH and from the Harare City Health Department.

The study methods have been described elsewhere [9]. Briefly, information on all births occurring from October 1, 1997, through September 30, 1998, at HMH was abstracted from the maternity delivery registry records. For each birth, information was abstracted on the date of birth, residential area of the woman (rural/urban), whether the mother attended prenatal care or not, maternal age and parity, estimated gestation, birth weight; sex and vital status of the baby at birth, whether the infant was a single or multiple delivery, and the type of delivery. It was not possible to link births from multiple gestations in this data set. A woman was considered to have received prenatal care when she had at least one visit for prenatal care during her pregnancy. Parity was the number of previous pregnancies ending after 20 completed weeks of gestation including stillbirth (categorized as 0, 1 to 2, and more than 2 pregnancies). Type of delivery denoted whether the infant was delivered vaginally presenting as cephalic, face to pubis or breech; vaginally with instrumental assistance; or by Cesarean section.

Eligibility criteria for this study were based on the WHO definition of viability, that is, a birth weight of at least 500 grams gestational age at least of 20 weeks [15]. Births without information on vital status were excluded. A stillbirth was defined as intrauterine death of a fetus weighing at least 500 grams after 20 completed weeks of gestation occurring before the complete expulsion or extraction from its mother. A fresh stillbirth was defined as the intrauterine death of a fetus during labor or delivery, and a macerated stillbirth was defined as the intrauterine death of a fetus sometime before the onset of labor, where the fetus showed degenerative changes [15] as reported in the obstetric records by the attending physician/midwife. An ENNM was defined as a death that occurred within the first hour of life.

Gestational age (GA) at birth was estimated by the number of days between the first day of the last menstrual period (LMP) and date of birth expressed in completed weeks after LMP and a clinical estimate as recorded in the maternity delivery record. Preterm birth was defined as a birth occurring at or before 37 completed weeks of gestation. A post-term birth was defined as a birth occurring after 44 weeks of gestation. Birth weight was defined as the first measurement of body weight, usually in the first hour of life, measured to the nearest gram. A LBW birth was defined as the birth of an infant weighing less than 2,500 grams at birth irrespective of gestational age. We also defined three LBW subgroups; term LBW birth, preterm LBW birth and very LBW birth defined as infants weighing below 1,500 grams. A high birth weight birth, based on the upper 10^th ^percentile of our birth weight distribution, was defined as the birth of an infant weighing above 3,500 grams.

A total of 18,149 births were recorded in the 12-month study period. On average between 50 to 60 deliveries occurred daily. We excluded 32 (0.2%) births below 20 weeks of gestation, 68 (0.4%) that did not have information on vital status, 78 (0.4%), that weighed below 500 grams at birth and 795 (4.4%) that were missing information on birth weight or estimated gestation. Finally we excluded an additional 103 (0.6%) births for which birth weight/gestational age combination were implausible based on the algorithm advocated by Alexander et al. [16], leaving 17,072 births for this analysis.

### Statistical analysis

The numbers of fresh, macerated, and un-typed stillbirths are presented as a proportion of all births, and ENNM are presented as a proportion of live births. To examine predictors of these outcomes, cross-tabulations by each covariate were examined using chi-square tests of homogeneity. As the complete population of births over a one-year period within the hospital was ascertained, we could directly estimate the risk of stillbirth and ENNM for each covariate. In the unadjusted analyses, relative risks (RR) and 95% confidence intervals (CI) were calculated for each demographic and reproductive risk factor using EP-INFO 2000. All 17,072 eligible births were included for stillbirth analyses. Given the differences in risk for singleton and multiple births, the subsequent multivariate analyses are limited to singleton births, therefore 16,023 singleton births were used for the stillbirths and combined stillbirth and ENNM analyses. For ENNM only analyses, 15,117 live singleton births were included.

Multivariable generalized linear regression models with a log-link function and binomial error using SAS [SAS Institute Inc., Cary, NC, USA] version 8.1, were used to model each outcome. A log (rather than logit) link function and binomial errors were used to allow estimation of relative risks (rather than odds ratios). Relative risks of stillbirth and 95% confidence intervals were calculated after adjusting for maternal age, residence, prenatal care, parity, infant sex and type of delivery. As estimates were not stable due to collinearity, we combined births delivered as face to pubis with those of normal vaginal delivery for the type of delivery analysis, adjusting for maternal age, residence, parity, and infant sex.

**Figure 1:**
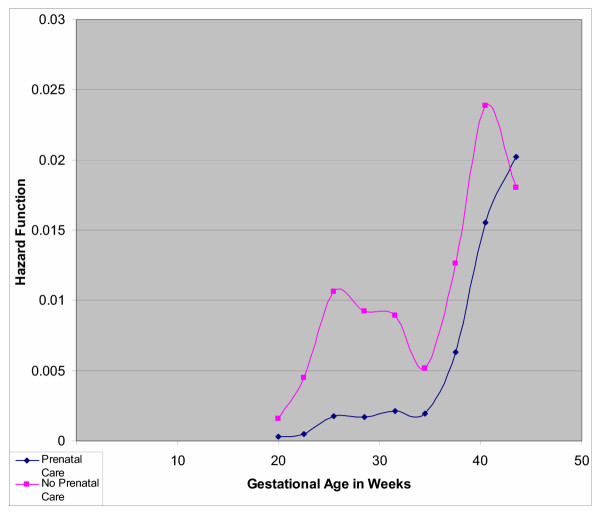
Probability of Combined Stillbirth and Early Neonatal Death among 16,023 Singleton Deliveries by Prenatal Care at Harare Maternity Hospital; October 1997 through September 1998

**Figure 2:**
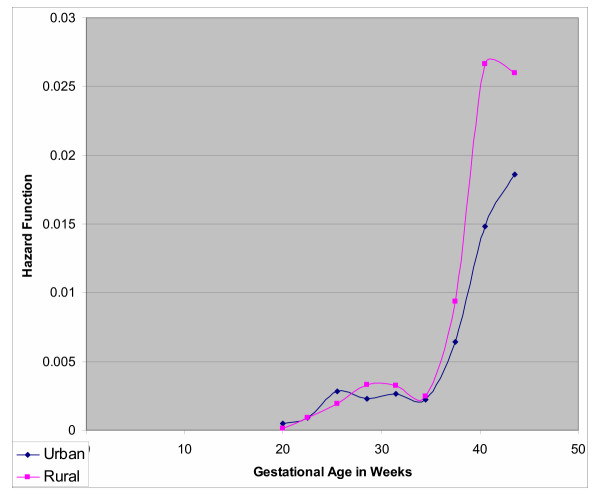
Probability of Death among 16,023 Singleton Deliveries by Residence at Harare Maternity Hospital; October 1997 through September 1998

Recent epidemiological literature has suggested at least two alternative methods of calculating the probability of stillbirth by GA, with the differences being in the definition of the denominator [17,18]. One method calculates the GA-specific probability of stillbirth as the number of stillbirths divided by the number of births at a given GA. A second method calculates the GA-specific hazard of stillbirth as the number of stillbirths at a given GA divided by the number of fetuses at the given GA still remaining to be born [17-19]. We provide estimates using the first definition in Tables 2 and Additional files 1 and 2, and use the second definition in Tables 3 and 4 and Figures 1 and 2. Covariate effects on gestational age-specific mortality were estimated using Cox regression model with estimated gestation at birth as the time variable. Hazard ratios (HR) for stillbirth and 95% CI were calculated [17,18]. All 16,023 singleton births were used for this analysis, and stillbirths and ENNM were combined as outcomes of interest [17,18]. The interaction between each covariate and time (GA) was tested for evidence of non-proportion hazards. Covariates with no evidence of non-proportion hazards (maternal age, infant sex and parity) were refitted without the interaction term. For covariates with evidence of non-proportion hazards (residence and prenatal care) we also calculated the risk of death at 20, 28, 32, 36, 40 and 42 weeks of gestation. The probabilities of death at each stage of GA were estimated using life tables for prenatal care and for urban versus rural residents, and are presented in Figures 1 and 2.

**Table 2:** Distribution and risk of Deaths within First Hour of Life for 15,117 Live, Singleton Deliveriesat Harare Maternity Hospital; October 1997 to September 1998

	**All Live Births**	**All Deaths Within First Hour of Life**		**Relative Risk (95% CI)**	**Adjusted ^a,b ^Relative Risk (95% CI)**
	**n**	**n**	**%**		
**Total**	15,117	129	0.9		

**Maternal age**					
Below 20	3,214	41	1.3	**1.69 (1.16 – 2.45)**	1.21 (0.77 – 1.89)
20 to 35	10,860	82	0.8	Reference	Reference
Above 35	969	4	0.4	0.55 (0.20 – 1.49)	0.57 (0.19 – 1.70)

**Infant sex**					
Male	7,594	85	1.0	**1.69 (1.17 – 2.45)**	**1.67 (1.15 – 2.43)**
Female	7,076	50	0.6	Reference	Reference

**Residence**					
Urban	13,915	106	0.8	Reference	Reference
Rural	2,118	22	1.0	1.27 (0.80–2.00)	1.36 (0.85 – 2.18)

**Prenatal care**					
At least one visit	13,527	101	0.8	Reference	Reference
No prenatal care	1,463	26	1.8	**2.38 (1.55 – 3.65)**	**2.52 (1.63 – 3.91)**

**Parity**					
Para 0	6,945	77	1.1	**1.81 (1.22 – 2.70)**	1.52 (0.97 – 2.38)
Para 1–2	5,723	35	0.6	Reference	Reference
Para above 2	2,377	15	0.6	1.03 (0.56 – 1.89)	1.25 (0.65 – 2.40)

**Delivery type**					
Normal vaginal delivery	11,658	82	0.7	Reference	Reference
Breech	386	27	7.0	**9.94 (6.52 – 15.18)**	**10.53 (6.78 – 6.34)**
Instrumental	344	9	2.6	**3.72 (1.88 – 7.34)**	**3.38 (1.64 – 6.96)**
Cesarean section	2,707	10	0.4	0.53 (0.27 – 1.01)	0.56 (0.29 – 1.09)

a For Adjusted analysis, maternal age, sex residence, prenatal care, parity were entered in one model

b Delivery type was adjusted for mother' age, residence, sex and parity

* Excludes 1,049 multiple gestations births, and 906 stillbirths

**Table 3:** Demographic and Obstetric Characteristics: Hazard Ratios of Combined Stillbirth and Early Neonatal Deaths for 16,023 Singleton Deliveriesat Harare Maternity Hospital; October 1997 to September 1998

	**Hazard Ratio (95% Confidence Intervals)**
**Maternal age**	
Below 20	0.86 (0.73 – 1.01)
20 to 35	Reference
Above 35	**1.50 (1.21 – 1.84)**

**Infant sex**	
Male	1.09 (0.96 – 1.24)
Female	Reference

**Parity**	
Para 0	0.91 (0.80 – 1.05)
Para 1–2	Reference
Para above 2	**1.20 (1.01 – 1.42)**

*There was no evidence of time dependence of mortality risk (i.e., no evidence of non-proportion hazards) for maternal age, infant sex, or parity

**Table 4:** Demographic and Obstetric Characteristics for which the Hazard Ratio Changed over Gestational Age, for Combined Stillbirth and Early Neonatal Deaths for 16,023 Singleton Deliveries at Harare Maternity Hospital; October 1997 to September 1998

	**Hazard Ratio (95% Confidence Interval)**				
**Gestational Age**	28	32	36	40	42

**Residence**					
Urban	Reference	Reference	Reference	Reference	Reference
Rural	1.04 (0.82 – 1.32)	1.22 (1.03 – 1.45)	1.44 (1.21 – 1.70)	1.69 (1.34 – 2.12)	1.83 (1.39 – 2.40)

**Prenatal care**					
At least one visit	Reference	Reference	Reference	Reference	Reference
No prenatal care	4.76 (4.00 – 5.66)	3.32 (2.84 – 3.87)	2.32 (1.90 – 2.82)	1.62 (1.23 – 2.11)	1.35 (0.99 – 1.84)

* There was evidence of time dependence of mortality risk for residence or receiving prenatal care

## Results

### Demographic and obstetric risk factors of stillbirth by type

Of the 959 stillbirths, 201 (21%) were fresh and 458 (48%) were macerated stillbirths; the type of stillbirth for 300 (31%) births could not ascertained from the available obstetrical records. The annual stillbirth ratio at HMH was 56 per 1,000 total births, of which 12 per 1,000 were fresh stillbirths, 27 per 1,000 were macerated stillbirths, and 17 per 1,000 were for stillbirths whose type was not indicated in the obstetric records (Table 1). Age of mothers ranged from 10 to 50 years. Most mothers were 20 to 35 years old, with a mean age at delivery of 24.6 years. Most mothers (86%) resided in urban areas. Women present late for prenatal care, GA less than 28 weeks accounted for 384 singleton births, of which 180 (47%) did not receive prenatal care. It is uncommon in Zimbabwe for women to present for prenatal care prior to 28 weeks of pregnancy [9,20], and in the rural areas women could present at a health center at the time of labor if they are having difficulty. About 45% of the women were primiparous, with parity ranging from 0 to 12. The mean age of mothers with parity of 0, was 20.7 (range 10–41 years), compared to 27.7 (range 14–50) for women with parity 1 and more. As expected, there were slightly more male than female births. A total of 17.5% of the deliveries were by Cesarean section, and an additional 2.2% required some form of instrumentation during delivery, while 5.9% of the births were either breech or face to pubis presentations. About 6% of the infants were from multiple gestations, and in crude analysis, multiple gestation births were less likely to be macerated stillbirths (RR = 0.57; 95% CI 0.35–0.93), but more likely to die in the first hour of life (RR = 1.88; 95% CI 1.12–3.14).

**Table 1:** Distribution of births by Vital Status at Harare Maternity Hospital; October 1997 to September 1998

	**Total Births**	**All Stillbirths**		**Fresh Stillbirths**		**Macerated Stillbirths**		**Un-typed Stillbirths**	
	**n**	**n**	**%**	**n**	**%**	**n**	**%**	**n**	**%**
**Total**	17,072	959	5.6	201	1.2	458	2.7	300	1.7

**Maternal age**									
Below 20	3,475	149	4.3	34	1.0	69	2.0	46	1.2
20 to 35	12,333	700	5.7	152	1.2	341	2.8	207	1.7
Above 35	1,177	102	8.7	15	1.3	44	3.7	43	3.7

**Infant sex**									
Male	8,546	462	5.4	91	1.1	226	2.6	145	1.7
Female	7,978	419	5.3	93	1.2	197	2.5	129	1.6

**Residence**									
Urban	14,576	794	5.5	170	1.2	391	2.7	233	1.6
Rural	2,402	163	6.8	31	1.3	65	2.7	67	2.8

**Prenatal care**									
At least one visit	15,135	729	4.8	158	1.1	353	2.3	218	1.4
No prenatal care	1,796	218	12.1	41	2.3	101	5.6	76	4.2

**Parity**									
Para 0	7,608	375	4.9	81	1.1	183	2.4	111	1.4
Para 1–2	6,563	383	5.8	76	1.1	189	2.9	118	1.8
Para above 2	2,821	195	6.9	42	1.5	85	3.0	68	2.4

**Gestation type**									
Singleton	16,023	906	5.7	189	1.2	441	2.8	276	1.7
Multiple	1,049	53	5.1	12	1.1	17	1.6	24	2.4

**Delivery type**									
Normal vaginal delivery	12,654	662	5.2	142	1.1	357	2.8	163	1.3
Breech	795	140	17.6	28	3.5	78	9.8	34	4.3
Instrumental	377	24	6.4	10	2.7	6	1.6	8	2.1
Cesarean section	2,990	92	3.1	9	0.3	10	0.3	73	2.5
Face to pubis	201	8	4.0	5	2.5	1	0.5	2	1.0

Additional file 1 presents the crude relative risks of stillbirth by type of demographic and reproductive characteristics of the study population. Young mothers were less likely than older women (RR = 0.73; 95% CI 0.61–0.88) to deliver a stillborn infant, with the reduction in risk particularly evident for macerated stillbirth (RR = 0.72; 95% CI 0.56–0.93). In contrast, women above 35 years had a 59% increased risk of stillbirth and 43% increase in the likelihood of delivering a macerated stillbirth. Rural women delivering at HMH had a 24% increased risk of stillbirth compared with women who resided in urban areas. Women who did not receive prenatal care consistently had over a 2.3-fold increase in the risk of stillbirth of any type. Compared to a normal vaginal delivery, breech deliveries were over 4.7 times more likely to be stillbirth, while births by Cesarean section were less likely to result in any type of stillbirth. Delivery by instrumentation was 2.2 times more likely to result in a fresh stillbirth than was normal vaginal delivery.

Additional file 2 presents adjusted relative risks for stillbirth by demographic and reproductive characteristics. Except for parity, which is correlated with maternal age (Pearson coefficient r = 0.76 p-value = 0.0001), the risks did not change in the adjusted analysis. Overall, risks for un-typed stillbirths did not differ from those for fresh and macerated stillbirths, except for residence and delivery by Cesarean section.

### Demographic and obstetric risk factors of ENNM

The early neonatal mortality ratio was 9 per 1000 live births. Table 2 presents the distribution and risk for ENNM by demographic and reproductive characteristics. Mothers under 20 years old were 69% more likely than mothers 20 to 35 years of age to deliver an infant who died within the first hour of life, and similarly, risk for primiparous women was 81% compared to multiparous women. Male infants had a 69% increased risk of dying within the first hour of life. Women who did not receive prenatal care consistently had over a 2.4-fold increase in the risk of ENNM. Compared to a normal vaginal delivery, breech deliveries were 9.9 times more likely to end up as ENNM. Delivery by instrumentation was 3.7 times more likely to result in ENNM than was normal vaginal delivery. Except for maternal age and parity the risks remained the same or elevated in adjusted analysis.

### Relationship of mortality to demographic and obstetric factors

Table 3 presents the crude hazard ratios for mortality (stillbirths and ENNM combined) for demographic and obstetric characteristics that had constant hazard ratios over GA. There was a 50% increased risk of death for mothers over age 35 years compared with mothers 20 to 35 years of age. There was a 20% increased risk of death for mothers parity above 2 compared with mothers with parity 1 to 2. Infant sex was not significantly associated with mortality.

### Relationship of mortality to prenatal care and residence

Table 4 presents crude hazard ratios for mortality (stillbirths and ENNM combined) for demographic and obstetric characteristics for which hazard ratios change over GA. These variables included prenatal care and residence. The hazard ratio for prenatal care decreased with increasing gestation, from 4.76 at 28 weeks to 1.35 at 42 weeks. Figure 1 depicts the hazard function by GA comparing mothers who did and did not receive prenatal care. At 20 weeks, the hazard functions for mothers who did not receive prenatal care and those who did are similar. However, because of the low probability of death before week 35 among those receiving prenatal care, the relatively higher probability among those not receiving prenatal care results in a high relative risk. After week 35, the mortality (stillbirths and ENNM combined) risk in both groups increases proportionally, but for those without prenatal care remain at higher risk and the effect is attenuated at 42 weeks.

As gestation increased, the risk of mortality for rural residence increased, from 1.04 at 28 weeks to 1.69 at 40 weeks and 1.84 at 42 weeks of gestation (Table 6). Figure 2 depicts the hazard function by gestational age comparing rural and urban residence. Up to 35 weeks the hazards are very similar, beyond 35 weeks, the hazards increase proportionally for both groups, but predominantly higher for births from mothers who resided in rural areas.

### Distribution of stillbirth and ennm by birth weight and ga categories

Additional file 3 shows the distribution of stillbirth and ENNM by birth weight and GA categories, using the traditional methods (birth weight-specific mortality, where LBW is analyzed as preterm LBW, term LBW) [18]. Sixteen percent of all stillbirths were LBW and 17% were preterm. Almost 3% of neonatal deaths were LBW, and 3% were preterm births.

## Discussion

This paper evaluates the distribution of and risk factors for fresh and macerated stillbirth and ENNM among mothers giving birth at the largest hospital serving Harare, Zimbabwe. The proportion of macerated stillbirths in Harare is higher than in more developed countries, suggesting the presence of insults to the developing fetus and the need for timely screening and management of chronic conditions and infections. A considerable proportion of the stillbirths were fresh stillbirths, and the frequency of ENNM was high, suggesting the need for improved obstetric care and availability of emergency services during the delivery period. Lack of prenatal care was associated with increased risk of stillbirth and ENNM whether we analyzed using traditional methods (gestational age-specific mortality) [18] or with GA as a time-varying factor as argued by other experts [21-23]. Similarly, rural residence was associated with increased risk of all stillbirth and ENNM whether we analyzed using traditional methods (gestational age-specific mortality) or with GA as a time-varying factor. But most importantly, using GA as a time-varying factor clarifies where and when the risk of death is more prominent. Fresh or macerated stillbirths and ENNM were more likely to be delivered breech, but less likely to be delivered by Cesarean section. Cesarean section appears to protect against stillbirth in this population. Fresh stillbirths and ENNM were also associated with delivery by instrumentation.

The incidence of stillbirth, 56 per 1,000 total births we report for HMH, is higher than the 26 per 1,000 total births reported by Iliff and colleagues using 1989 data from HMH and 9 Harare municipal clinics [12,13], and higher than the 45 per 1,000 live births at Mpilo Maternity Hospital [24], another large referral hospital in the second largest city in Zimbabwe. Our findings differ from previous Zimbabwean studies because HMH is the largest referral center in this country, and would be expected to have higher mortality rates than other hospitals referring their most complicated cases. When we recalculate our rates based on the number of deliveries in the GHMU, 56% of which occur at HMH [9,25], and assuming no stillbirths occurred in the clinics, we estimate a population-based stillbirth ratio of 33 per 1,000 total births, a figure more comparable to that reported by Iliff and colleagues.

In this population, more stillbirths were macerated, suggesting existence of problems linked to the antenatal period, which could be related to congenital malformations [2,4]; obstetric hemorrhage; preclampsia [2,4-6,26]; infections such as syphilis [7,8,26,27]; or existing maternal chronic conditions such as hypertension, cardiac disease, and diabetes [2,4-6], none of which our study had the ability to evaluate. Smoking, which is an important factor especially in developed countries, was not a factor for this population [28]. Fresh stillbirths contribute 1.2% of all births while ENNM contribute 0.9% of live births at this institution, and both are likely to be related to fetal hypoxia [2,12,26], congenital malformations [2,4,12], quality of delivery care given to a woman during labor and delivery, and poor access to emergency obstetric care.

As would be expected, lack of prenatal care was consistently and strongly associated with stillbirths and ENNM, similar to what other studies have reported [7,29-31]. Although women in Zimbabwe usually begin prenatal care at 28 weeks of gestation a considerable number will present at hospital before that time for a problem related to their pregnancy leading to an adverse birth outcome [[9,20], and [28]]. Had the adverse birth outcome not occurred prior to 28 weeks of gestation, these women could have had an opportunity to present for prenatal care, later in their pregnancy as is common for most women in Zimbabwe. The risk of mortality (stillbirths and ENNM combined) increases with increasing GA before 35 weeks of gestation, but decreases proportionally thereafter. There was a crossover of risk of mortality by prenatal care much later in the course of pregnancy, at 42 weeks of gestation, a phenomenon reported but at earlier gestational age by other studies [17-19]. Thus, the risk of mortality was much higher for women who did not receive prenatal care compared to those who did in the earlier gestational ages, and was moderately higher proportionally after 35 weeks of gestation, and was attenuated at term. In developing countries, prenatal care, even if only attended once, remains an important factor in obstetric care, as this may be a critical linkage between the woman with maternity care services [9,28]. In contrast, research findings in middle-income countries emphasize the importance of the number of prenatal care visits and the adequacy and quality of prenatal care services. WHO recommends using prenatal care as a strategy for improved obstetric care [32]. Our data suggest that prenatal care may help ensure that interventions occur in a timely manner.

Rural residence was associated with increased risk of all stillbirths as reported by previous studies [9,28]. The risk of mortality (stillbirths and ENNM combined) increased with increasing GA, similar to other study reports [17-19]. Before 35 weeks both rural and urban women have a similar risk of mortality. Although the risk of mortality increases proportionally to term for both groups, rural women have a higher risk of having their infant dying in utero and within the first hour of life. Prior to 28 weeks a considerable number of women from rural residence did not receive prenatal care, 39 (10%). After 35 weeks rural women who end up with their infant dying in utero or within the fist hour of life might have been women referred to HMH from rural centers with a condition/complication related or leading to the adverse birth outcome. Caution should be taken when interpreting this finding, because we have two artifacts. Intuitively, after 35 weeks, women who did not receive prenatal care are primarily from rural areas and were likely not to receive prenatal care throughout their pregnancy. Secondly, we do not have the counter population (rural women who attended prenatal care) in our denominator.

For maternal age, infant sex and parity in our study, which were not time-dependent, using either traditional methods (gestational age-specific mortality) [18] or the analysis where GA is a time varying factor did not change the risk of mortality (stillbirths and ENNM combined). But, using GA as a time-varying factor for the analysis helps us to understand further the relationship between some of the maternal factors and mortality, which otherwise would have been missed. For prenatal care and residence, we were able to show how the risk of mortality was distributed at each stage of GA. We were able to separate effects at early periods versus later stages in pregnancy, which is useful for health-care planners, policy makers, and implementers, in terms of targeting resources. For example, for prenatal care, we were able to show that the risk of morality is high at early gestation. The risk of mortality persists later in pregnancy, but decreasing proportionally throughout pregnancy, being higher for women who did not receive prenatal care. This finding suggests a need to focus and emphasize on early booking and the critical role of prenatal care in developing countries. With regards to residence, knowledge that infants of rural women who get referred to urban institutions have the highest risk of mortality may suggest the need to pay more attention during the antenatal period and to improve the referral system and emergency care services.

Stillbirths, irrespective of type, and ENNM were less likely to be delivered by Cesarean section. It is conceivable that factors leading to stillbirth may cause mothers to have a Cesarean section, but our results show that Cesarean section was consistently protective of either stillbirth or ENNM. This finding may suggest that obstetricians are careful not to perform Cesarean section unless it is indicated for stillbirths, or ENNM, or that whenever a Cesarean section is performed, it saves life of the infant.

Stillbirths and ENNM were likely to be delivered breech. For fresh stillbirths and ENNM, this finding is consistent with the clinical observation that because of the nature and dynamics of this type of delivery, these infants are likely to die during or at delivery [9,28]. Similarly, infants delivered by some form of instrumentation were more likely to die within the first hour of life. For macerated stillbirths, this finding may be more related to preterm infants and would be consistent with the clinical observation that the infants turn to optimal birth presentation at about 34 weeks of gestation. About 242 (47%) of singleton births delivered as breech were preterm. Intuitively, infants that are at risk because of their small size and level of maturity are likely to face the additional risk of breech presentation.

Maternal age effects were common in stillbirths, consistent with other studies [33-35]. But the effects of maternal age were more prominent for macerated versus fresh stillbirths, again strengthening the possibility that maternal chronic disease conditions in later years of life may play a significant role. Additionally, older mothers were at greater risk for stillbirth, but lower risk for neonatal death. The crude risk for young maternal age, which was similar to crude risk for primiparity for early neonatal births, was attenuated after controlling for residence, parity, prenatal care and infant sex.

This study has some limitations. As the study was a retrospective analysis of data obtained from delivery logs, we were unable to examine risk factors such as chronic and comorbid conditions, congenital malformations, obstetric complications, and infections. Although we could not identify the stillbirth status of nearly one-third of stillbirths, risk estimates for un-typed stillbirths were similar to those for fresh and macerated stillbirths. Arguably, focusing solely on births within HMH raises concerns about selection bias. However, when we adjust our estimated rate to the base population, our rates are comparable to those previously reported [9,12-14]. Information on gestational age was limited to the clinicians' estimate and LMP information reported as weeks recorded in the obstetric log, thus some error in the classification of preterm births is likely [36,37]. Regardless, this pilot study is one of the few to characterize socio-demographic and reproductive risk factors for stillbirth and ENNM in this population [9]. Our ability to distinguish risks for macerated and fresh stillbirth has direct implications on quality of care given to pregnant women in Zimbabwe.

We were not able to show risk by combined birth weight and GA categories, which would otherwise be important for clinicians [17-19], [38,39], for to do so is impossible as birth weight varies with GA [17]. Therefore, it would be not feasible to put both variables in the same model. Our use of GA as a time-varying variable in this analysis helps conceptualize and define where risk occurs in the GA continuum.

## Conclusion

Our findings suggest that earlier perinatal care could assist in early identification and treatment of risk factors for macerated stillbirth, especially those that are preventable. Zimbabwean women enter prenatal care late in pregnancy, booking at 28 weeks or later [9,29-31]. Effective programs to decrease the frequency of stillbirth may require that entry to prenatal care begin by at least 20 weeks of gestation. Increased focus on health education programs, which emphasize the benefits of prenatal care and early booking in the first trimester or by 20 weeks of pregnancy, is needed. Earlier booking for prenatal care creates a critical linkage between the woman and the health care system, which may increase the probability that the woman will seek emergency care in a timely manner. In Zimbabwe, more focus is needed on the timing and adequacy of care in maternal and child health programs, and more research is needed on barriers to early entry to prenatal care.

There is also a need to improve quality of care and access to emergency care during labour and delivery to reduce the number of fresh stillbirths and ENNM. Cesarean section should be made readily available as it improves birth outcomes. Further studies should incorporate information from women served by the entire GHMU, to improve infant mortality and morbidity in Zimbabwe.

## Competing interests

The author(s) declare that they have no competing interests.

## Authors' contributions

SF conceived of the study, participated in design of the study, carried out the data collection and all analyses and drafted the manuscript.

SD participated in conceiving and designing the study, directed the analysis of the study and reviewed the manuscript.

KW participated in the analysis of the study and formulated the probability functions used to construct the figures.

BG participated in the analysis of the study, reviewed models to calculate the relative risk, and reviewed the manuscript. All authors read and approved the final manuscript.

## Pre-publication history

The pre-publication history for this paper can be accessed here:



## Supplementary Material

Additional File 1 Demographic and Obstetric Characteristics and Crude Risks of Stillbirth for 16,023 Singleton Deliveries at Harare Maternity Hospital; October 1997 to September 1998 Click here for file

Additional File 2 Adjusted^a ^Demographic and Obstetric Characteristics and Risks of Stillbirth for 16,023 Singleton Deliveries at Harare Maternity Hospital; October 1997 to September 1998 Click here for file

Additional File 3 Frequency of Stillbirth by Birth Weight and Gestational Age Categories for 17,072 Deliveries at Harare Maternity Hospital; October 1997 to September 1998 Click here for file
