# Supplementary material for: Incidence of stillbirth and perinatal mortality and their associated factors among women delivering at Harare Maternity Hospital, Zimbabwe: a cross-sectional retrospective analysis
Source: BMC Pregnancy Childbirth. 2005 May 5;5:9. doi: 10.1186/1471-2393-5-9 (PMC1156907; doi:10.1186/1471-2393-5-9)
Supplement: Additional File 1 — Demographic and Obstetric Characteristics and Crude Risks of Stillbirth for 16,023 Singleton Deliveries at Harare Maternity Hospital; October 1997 to September 1998 [file 1471-2393-5-9-S1.doc]

**Additional File 1. Demographic and Obstetric Characteristics and Crude Risks of Stillbirth for 16,023 Singleton Deliveries**

**at Harare Maternity Hospital; October 1997 to September 1998**

|  | **All Stillbirths**  **RR (95% CI)** | **Fresh Stillbirths**  **RR (95% CI)** | **Macerated Stillbirths**  **RR (95% CI)** | **Un-typed Stillbirths**  **RR (95% CI)** |
| --- | --- | --- | --- | --- |
| **Maternal age**  Below 20  20 to 35  Above 35 | **0.73 (0.61 – 0.88)**  Reference  **1.59 (1.30 – 1.95)** | 0.68 (0.46 – 1.01)  Reference  1.16 (0.68 – 1.96) | **0.72 (0.56 – 0.93)**  Reference  **1.43 (1.04 – 1.95)** | 1.30 (0.93 – 1.80)  Reference  **2.32 (1.66 – 3.24)** |
| **Infant sex**  Male  Female | 1.03 (0.90 – 1.18)  Reference | 0.97 (0.72 – 1.30)  Reference | 1.06 (0.88 – 1.29)  Reference | 1.03 (0.81 – 1.32)  Reference |
| **Residence**  Urban  Rural | Reference  **1.24 (1.05 – 1.47)** | Reference  1.10 (0.75 – 1.64) | Reference  1.06 (0.82 – 1.37) | Reference  **1.71 (1.29 – 2.26)** |
| **Prenatal care**  At least one visit  No prenatal care | Reference  **2.54 (2.19 – 2.94)** | Reference  **2.32 (1.63 – 3.30)** | Reference  **2.63 (2.12 – 3.27)** | Reference  **2.96 (2.26 – 3.88)** |
| **Parity**  Para 0  Para 1-2  Para above 2 | **0.82 (0.71 – 0.95)**  Reference  **1.21 (1.01 - 1.44)** | 0.85 (0.61 – 1.17)  Reference  1.36 (0.93 – 2.00) | 0.83 (0.68 – 1.02)  Reference  **1.34 (1.04 - 1.73)** | 0.77 (0.59 – 1.01)  Reference  1.31 (0.96 - 1.78) |
| **Delivery type ^a^**  Normal vaginal delivery  Breech  Instrumental  Cesarean section | Reference  **4.74 (4.00 – 5.61)**  0.96 (0.61 – 1.51)  **0.63 (0.50 – 0.78)** | Reference  **5.36 (3.56 – 8.05)**  **2.16 (1.11 – 4.28)**  **0.28 (0.14 – 0.55)** | Reference  **5.36 (4.23 – 6.80)**  0.59 (0.27 – 1.32)  **0.13 (0.07 – 0.24)** | Reference  **5.43 (3.69 – 7.98)**  0.67 (0.22 – 2.10)  **2.01 (1.53 –2.66)** |

a We combined infants born face to pubis with normal vaginal delivery as estimates were unstable

* Excludes 1,049 multiple gestation births

** Abbreviations: RR = relative risk; CI = confidence intervals.
