# Supplementary material for: Incidence of stillbirth and perinatal mortality and their associated factors among women delivering at Harare Maternity Hospital, Zimbabwe: a cross-sectional retrospective analysis
Source: BMC Pregnancy Childbirth. 2005 May 5;5:9. doi: 10.1186/1471-2393-5-9 (PMC1156907; doi:10.1186/1471-2393-5-9)
Supplement: Additional File 2 — Adjusteda Demographic and Obstetric Characteristics and Risks of Stillbirth for 16,023 Singleton Deliveries at Harare Maternity Hospital; October 1997 to September 1998 [file 1471-2393-5-9-S2.doc]

**Additional File 2. Adjusted ^a^ Demographic and Obstetric Characteristics and Risks of Stillbirth for 16,023 Singleton Deliveries at Harare Maternity Hospital; October 1997 to September 1998**

|  | **All Stillbirths**  **RR (95% CI)** | **Fresh Stillbirths RR (95% CI)** | **Macerated Stillbirths**  **RR (95% CI)** | **Un-typed Stillbirths**  **RR (95% CI)** |
| --- | --- | --- | --- | --- |
| **Maternal age**  Below 20  20 to 35  Above 35 | **0.73 (0.59 – 0.89)**  Reference  **1.46 (1.12 – 1.89)** | 0.79 (0.50 – 1.24)  Reference  0.85 (0.45 – 1.61) | **0.70 (0.52 – 0.94)**  Reference  **1.48 (1.00 – 2.18)** | 0.76 (0.52 – 1.12)  Reference  **2.11 (1.33 – 3.33)** |
| **Infant sex**  Male  Female | 1.04 (0.91 – 1.18)  Reference | 1.00 (0.74 – 1.34)  Reference | 1.07 (0.88 – 1.30)  Reference | 1.03 (0.80 – 1.32)  Reference |
| **Residence**  Urban  Rural | Reference  **1.33 (1.12 – 1.59)** | Reference  1.14 (0.75 – 1.73) | Reference  1.15 (0.88 – 1.51) | Reference  **1.77 (1.31 – 2.39)** |
| **Prenatal care**  At least one visit  No prenatal care | Reference  **2.66 (2.28 – 3.11**) | Reference  **2.43 (1.68 – 3.51)** | Reference  **2.75 (2.19 – 3.46)** | Reference  **2.80 (2.71 – 4.76)** |
| **Parity**  Para 0  Para 1-2  Para above 2 | 0.93 (0.79 – 1.09)  Reference  1.02 (0.83 - 1.26) | 0.91 (0.63 – 1.32)  Reference  1.41 (0.91 – 2.20) | 0.97 (0.77 – 1.22)  Reference  0.95 (0.69 - 1.31) | 0.87 (0.64 – 1.18)  Reference  0.89 (0.60 – 1.33) |
| **Delivery type ^bc^**  Normal vaginal delivery Breech  Instrumental  Cesarean section | Reference  **4.65 (3.88 – 5.57)**  0.84 (0.50 – 1.41)  **0.64 (0.51 – 0.79)** | Reference  **5.47 (3.60 – 8.33)**  1.95 (0.91 – 4.15)  **0.29 (0.15 – 0.57)** | Reference  **5.13 (3.98 – 6.59)**  0.64 (0.29 – 1.43)  **0.13 (0.07 – 0.24)** | Reference  **5.38 (3.55 – 8.17)**  0.26 (0.04 – 1.86)  **2.17 (1.62 –2.80)** |

a For adjusted analysis for all types of stillbirths, maternal age, sex residence, prenatal care, parity were entered in one model for each outcome

b Delivery type was adjusted for mother’ age, residence, sex and parity for all stillbirths, fresh stillbirths, and un-typed stillbirths

c We combined infants born face to pubis with normal vaginal delivery as estimates were unstable

* Excludes 1,049 multiple gestation births

** Abbreviations: RR = relative risk; CI = confidence intervals.
