# Supplementary material for: Incidence of stillbirth and perinatal mortality and their associated factors among women delivering at Harare Maternity Hospital, Zimbabwe: a cross-sectional retrospective analysis
Source: BMC Pregnancy Childbirth. 2005 May 5;5:9. doi: 10.1186/1471-2393-5-9 (PMC1156907; doi:10.1186/1471-2393-5-9)
Supplement: Additional File 3 — Frequency of Stillbirth by Birth Weight and Gestational Age Categories for 17,072 Deliveries at Harare Maternity Hospital; October 1997 to September 1998 [file 1471-2393-5-9-S3.doc]

**Additional File 3**. Frequency of Stillbirth by Birth Weight and Gestational Age Categories for 17,072 Deliveries at Harare Maternity Hospital; October 1997 to September 1998

|  | **Total Births**  **n** | **All Stillbirths**  **n %** | | **Fresh Stillbirths**    **n %** | | **Macerated Stillbirths**    **n %** | | **Unidentified Stillbirths**    **n %** | | **Live Births**  **n** | **Deaths Within First Hour of Life**  **n %** | |
| --- | --- | --- | --- | --- | --- | --- | --- | --- | --- | --- | --- | --- |
| All births | 17,072 | 959 | 5.6 | 201 | 1.2 | 458 | 2.7 | 300 | 1.8 | 16,113 | 145 | 0.9 |
| **Birth weight categories by gestational age** |  |  |  |  |  |  |  |  |  |  |  |  |
| Term normal birth weight ^a^ | 10,368 | 243 | 2.3 | 83 | 0.8 | 86 | 0.8 | 74 | 0.7 | 10,077 | 48 | 0.5 |
| Term <2500 grams | 1,589 | 118 | 7.4 | 30 | 1.9 | 46 | 2.9 | 42 | 2.6 | 1,471 | 10 | 0.7 |
| Term >3500 grams | 1,837 | 48 | 2.6 | 10 | 0.5 | 16 | 0.9 | 22 | 1.2 | 1,789 | 9 | 0.5 |
| Preterm ≥2500 grams | 979 | 61 | 6.2 | 13 | 1.3 | 24 | 2.5 | 24 | 2.5 | 918 | 4 | 0.4 |
| Preterm <2500 grams | 2,275 | 489 | 21.5 | 65 | 2.9 | 286 | 12.6 | 138 | 6.1 | 1,786 | 74 | 4.1 |
| Preterm <1500 grams | 938 | 323 | 34.4 | 41 | 4.4 | 192 | 20.5 | 90 | 9.6 | 615 | 66 | 10.7 |
|  |  |  |  |  |  |  |  |  |  |  |  |  |
| **All births <2500 grams ^b^** | 3,864 | 607 | 15.7 | 95 | 2.5 | 332 | 8.6 | 180 | 4.7 | 3,257 | 84 | 2.6 |
|  |  |  |  |  |  |  |  |  |  |  |  |  |
| Gestational age categories |  |  |  |  |  |  |  |  |  |  |  |  |
| Term births ^c^ | 13,785 | 408 | 3.0 | 123 | 0.9 | 147 | 1.1 | 140 | 1.0 | 13377 | 67 | 0.5 |
| All preterms <37 weeks | 3,254 | 550 | 16.9 | 78 | 2.4 | 310 | 9.5 | 162 | 5.0 | 2,704 | 78 | 2.9 |
| ≥32 to <37 weeks | 2,150 | 205 | 9.5 | 31 | 1.4 | 103 | 4.8 | 71 | 3.3 | 1,945 | 13 | 0.7 |
| ≥28 to <32 weeks | 661 | 170 | 25.7 | 28 | 4.2 | 104 | 15.7 | 38 | 5.8 | 491 | 18 | 3.7 |
| ≥20 to <28 weeks | 443 | 175 | 39.5 | 19 | 4.3 | 103 | 23.3 | 53 | 12.0 | 268 | 47 | 17.5 |

a Reference group for all birth weight comparisons includes births ≥2500 to ≤3500 grams.

b Includes term and preterm births <2500 grams.

c Reference group for all gestation comparisons includes all births (of total births) ≥37 to <45 weeks.
